# Three New Mutations and Mild, Asymmetrical Phenotype in the Highly Distinctive LAMM Syndrome: A Report of Eight Further Cases

**DOI:** 10.3390/genes10070529

**Published:** 2019-07-12

**Authors:** Amina Al Yassin, Felice D’Arco, Matías Morín, Waheeda Pagarkar, Katherine Harrop-Griffiths, Azhar Shaida, Elena Fernández, Tom Cullup, Bianca De-Souza, Miguel Angel Moreno-Pelayo, Maria Bitner-Glindzicz

**Affiliations:** 1 North East Thames Regional Genetics Service, Great Ormond Street Hospital for Children NHS Foundation Trust, London WC1N 3JH, UK; 2 Department of Radiology, Great Ormond Street Hospital for Children NHS Foundation Trust, London WC1N 3JH, UK; 3 Servicio de Genética, Instituto Ramón y Cajal de Investigación Sanitaria (IRYCIS) and Hospital Universitario Ramón y Cajal, CIBERER, 28034 Madrid, Spain; 4 North West Thames Regional Genetic Service, London North West Hospitals NHS Trust, Harrow, Middlesex HA1 3UJ, UK; 5 Nuffield Hearing and Speech Centre, Royal National Throat Nose and Ear Hospital, 330 Grays Inn Road, London WC1X 8DA, UK; 6 ENT Department, Royal National Throat Nose and Ear Hospital, 330 Grays Inn Road, London WC1X 8DA, UK; 7 Otorhinolaryngology Department, San Cecilio Universitary Hospital, 18016 Granada, Spain; 8 Genetics and Genomic Medicine Programme, UCL Great Ormond Street Institute of Child Health, 30 Guilford St, London WC1N 1EH, UK

**Keywords:** *FGF3*, LAMM syndrome, labyrinthine aplasia, microtia, and microdontia, congenital deafness, external ear abnormalities

## Abstract

Labyrinthine aplasia, microtia, and microdontia (LAMM) is an autosomal recessive condition causing profound congenital deafness, complete absence of inner ear structures (usually Michel’s aplasia), microtia (usually type 1) and microdontia. To date, several families have been described with this condition and a number of mutations has been reported. We report on eight further cases of LAMM syndrome including three novel mutations, c. 173T>C p.L58P; c. 284G>A p.(Arg95Gln) and c.325_327delinsA p.(Glu109Thrfs*18). Congenital deafness was the primary presenting feature in all affected individuals and consanguinity in all but two families. We compare the features in our patients to those previously reported in LAMM, and describe a milder, asymmetrical phenotype associated with *FGF3* mutations.

## 1. Introduction

Labyrinthine aplasia, microtia, and microdontia (LAMM) syndrome is one of over 400 syndromes that cause deafness [1]. It was first described by Tekin et al. in 2007 and to date, over 56 individuals from 13 unrelated families have been reported [2]. It is recessively inherited and was originally described as causing labryinthine aplasia, (complete absence of inner ear structures also called Michel’s aplasia), microtia (usually type 1), and microdontia with small widely spaced teeth. Michel’s aplasia refers to complete membranous aplasia of the inner ear with or without presence of the otic capsule and/or petrous bone; it is due to a developmental arrest before the formation of the otocyst probably before the third week of development [3].

Subsequent reports described asymmetrical features, some inner ear formation (rudimentary otocysts), and normal looking auricles/external ears. Type 1 microtia (affecting the upper pinna) is a common feature, but again has been variable between cases. Cognitive abilities are intact but affected people have motor delay, attributed to absence of vestibular organs. Microdontia has been a consistent feature described in all the cases so far.

LAMM is caused by mutations in the *FGF3* gene [2] known to be important for the formation of the inner ear in mouse and chick [4,5]. Inner ear development starts when surface ectoderm adjacent to the hindbrain is induced to thicken and form a placode; this invaginates to form the otic vesicle which subsequently undergoes differentiation and morphogenesis giving rise to the complex compartments and structures observed in the mature inner ear. However, *Fgf3*-null mouse models undergo normal otic vesicle formation [4,5], but develop variable and incompletely penetrant inner ear malformations involving dorsal otic patterning [6]. FGF3 is also required for the development of the tooth stellate reticulum cells (dental core) which may be why microdontia is a common feature [7].

Mutations observed in LAMM patients include missense, nonsense and small frameshifts suggesting that LAMM syndrome is caused by loss of FGF3 function although functional studies have not been done [1].

We describe eight further cases from five unrelated families, one of them being the third family of European origin, with features of LAMM including three novel mutations. Whilst we also found mutations in asymmetrical and milder cases recapitulating the variable expressivity seen in mouse models lacking both FGF3 and FGF10, we failed to show that lack of FGF3 can cause unilateral deafness and inner ear malformation.

## 2. Patients and Methods 

Patients and affected and healthy relatives were recruited from public hospitals from University Hospital Ramón y Cajal (Madrid-Spain) and Great Ormond Street Hospital for Children NHS Foundation Trust (London-UK). Genomic DNA was obtained from peripheral blood using standard procedures. This study was designed in compliance with the tenets of the Helsinki Declaration, and patient enrolment was approved by the ethics committees of both institutions (E.A code 288-17, 24/August/2018). All participants or their legal guardians provided written informed consent prior to their participation in this study. Patients 1, 2, 4, 5, 6 and 8 were subject to Sanger sequencing. Patient 7 was tested on a clinical exome (Agilent SureSelect Focussed Exome Plus 1), with sequencing conducted on a NextSeq500 (Illumina, San Diego, CA, USA), and interrogated for a virtual panel of genes associated with LAMM syndrome and related disorders (*FGF3*, *EYA1*, *SIX1*). Variants identified on the virtual panel were confirmed by Sanger sequencing and parental segregation studies confirmed that the two variants were on opposite alleles. Two additional patients with unilateral cochlear malformations were tested by Sanger sequencing of coding exons and intron/exon boundaries of *FGF3*. Clinical details of patients presented here are summarized in Table 1. Comparison with previously reported cases is made in Table 2.

## 3. Results

### 3.1. Clinical Description of Patients

#### 3.1.1. Patients 1 and 2

**Figure 1:**
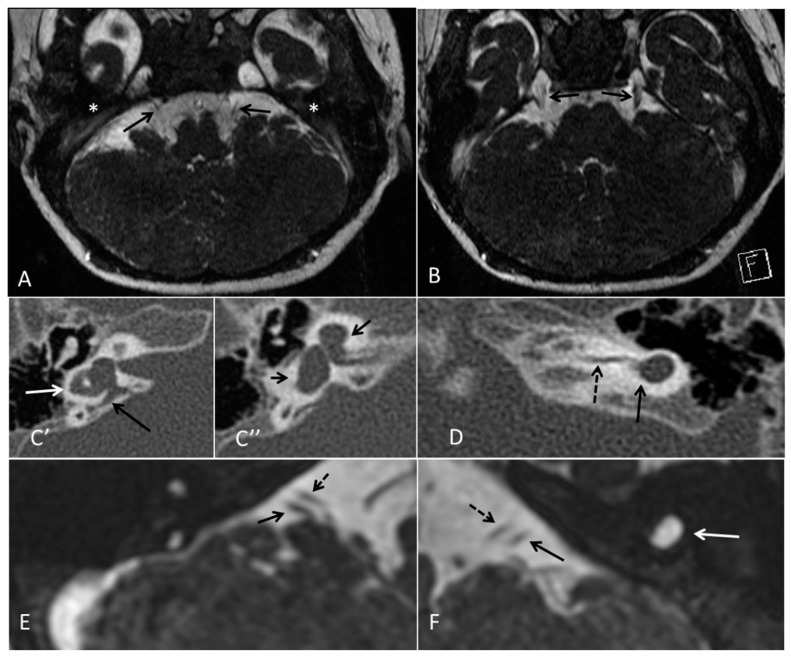
Axial T2 high resolution 3D sequence on the internal auditory canal (IAC) in patient 1 (**A**,**B**) show absence of membranous labyrinth in both petrous bones (*) with presence of VII nerves (arrows in **A**) and V nerves (arrows in **B**). Axial CT petrous bone in patient 7 shows incomplete partition type 1 malformation on the right (**C′**,**C″**), characterised by dysplastic lateral semicircular canal (white arrow in **C′**), dilated vestibular aqueduct (black arrow in **C′**), dilated cochlea and vestibule without visualisation of the modiolus (arrows in **C″**). In the same patient on the left (**D**) there is a rudimentary otocyst (arrow) with very narrowed IAC (dotted arrow). Axial T2 high resolution 3D sequence on the IAC in patient 7 (**E**,**F**) demonstrates presence of VII (dotted arrow) and VIII (black arrow) cranial nerves in the ponto-cerebellar angle on both sides. The rudimentary otocyst on the left is also noted (**F**, white arrow).

Patient 1, is the first child of consanguineous Asian parents, induced at 37 weeks for Intrauterine Growth Restriction (IUGR) and oligohydramnios. Birthweight was 1.98 kg but she required no support afterwards. She failed newborn hearing screening; brainstem auditory responses were absent at maximum level. She sat unsupported at 8 months and walked at 2 years. She has bilateral preauricular skin tags, cupped pinnae and small, widely spaced teeth. She has a shallow sacral pit. MRI with specific T2 3D sequences on the inner ears demonstrated absent cochleae, VIII nerves and vestibular structures while the VII and V nerves were visualised (Figure 1A,B). She has a history of two febrile seizures; on ultrasound scanning her right kidney was 8 mm smaller than left, but within normal limits. She uses British Sign Language (BSL). Her younger sibling, patient 2, was born at 37 weeks following induction of labour for IUGR and oligohydramnios; her birthweight was >2 kg. She failed newborn hearing screening and was found to be profoundly deaf. She sat by 9 months and walked at 2.5 years. On last examination at 2.5 years she was very unsteady and had a broad-based gait. She has cup- shaped, simple, protruding ears with no tags. Teeth are small and widely spaced. Imaging revealed absence of inner ear structures. She communicates by BSL although her communication is delayed and a social communication disorder is being considered. Both girls are homozygous for a novel mutation in *FGF3*: c.325_327delinsA (p.Glu109Thrfs*18), confirmed with bidirectional Sanger sequencing. The parents are heterozygous carriers.

#### 3.1.2. Patient 3

Patient 3 is a 42 year old Yemeni man born to first cousin parents. He has two sisters and a maternal grandmother with deafness and small teeth. There is consanguinity in previous generations of the family. His two children are unaffected and he is unrelated to his wife who is hearing. He has been profoundly deaf since birth and is a BSL user. He has experienced progressive disequilibrium and balance problems. He had past rhinitis and sinus surgery. He has a tiny preauricular skin hillock on one side; his external ears are otherwise normal. His teeth are small. CT of petrous temporal bones showed bilateral Michel’s aplasia with no visible inner ear structures or internal auditory canal (IAC), and hypoplastic deformed petrous pyramids. He has a homozygous mutation, c.283C>T (p.Arg95Trp), which has been reported previously [9,11].

#### 3.1.3. Patient 4, 5 and 6

These three affected siblings are children of first cousin Somalian parents. There are six unaffected siblings. The eldest affected sibling, Patient 4, was delivered normally at 42 weeks. She did not have newborn hearing screening and was confirmed to have profound deafness at 3 years. She sat at 8 months and walked at 18 months. She uses BSL. On examination she has cup-shaped ears with an upper pinna defect requiring surgical correction. She has small widely-spaced teeth with gum hypertrophy. MRI showed absent inner ears with a minimal cochlear rest on the left and total cochlear aplasia on the right. A large arachnoid cyst was also noted. She has type 1 diabetes mellitus, glasses for long-sightedness and asymmetrical kidneys (7.7/9.1 cm) which are within normal limits. Patient 5, was born vaginally at 42 weeks. He failed newborn hearing screening and is profoundly deaf. He sat at 8 months and walked at 18 months. He is a BSL user. On examination he had bilateral prominent ears with lop ear deformity (the top 1/3 of the pinna was deficient requiring bilateral surgical pinnaplasty) and widely-spaced small teeth. MRI showed bilateral Michel’s aplasia. He wears glasses for long-sightedness. Patient 6, is the youngest child of nine, born by normal delivery at term. She achieved head control fully at 9 months, sat unsupported at 10 months and was not walking when examined at 15 months. She uses BSL. She has protruding lop ears. She also has microdontia. MRI of the inner ears showed no inner ear on the left and rudimentary otocyst on the right. She has a left sided convergent squint and a history of obstructive sleep apnoea. Patients 4 and 5 are homozygous for c.283C>T (p.Arg95Trp); the parents declined testing for patient 6.

#### 3.1.4. Patient 7

Patient 7 is the 2-year old son of non-consanguineous Indian parents, born at 37 weeks. He failed newborn hearing screening and has severe-profound sensorineural hearing loss. He sat at 8 months but was not walking unsupported at 2 years. Renal scan was normal. The father has mild 4Hz hearing loss unilaterally. He has large prominent ears with preauricular ear tags on the right and widely- spaced teeth. Axial CT petrous bone in patient 7 (Figure 1C,D) shows incomplete partition type 1 malformation on the right (C′ and C″), characterised by dysplastic lateral semicircular canal (white arrow in C′), dilated vestibular aqueduct (black arrow in C′), dilated cochlea and vestibule without visualisation of the modiolus (arrows in C″). In the same patient on the left (D) there is a rudimentary otocyst (arrow) with very narrowed IAC (dotted arrow). Axial T2 high resolution 3D sequence on the IAC in patient 7 (Figure 1E,F) demonstrates presence of VII (dotted arrow) and hypoplastic VIII (black arrow) cranial nerves in the ponto-cerebellar angle on both sides. The rudimentary otocyst on the left is also noted (white arrow). He subsequently had a failed cochlear implant and a failed auditory brain stem implant on the right. He is being considered for a left auditory brainstem implant. He is compound heterozygous for a novel mutation c.284G>A (p.Arg95Gln) and c.534C>G (p.Phe178Leu) as previously described in a family with LAMM syndrome [14].

#### 3.1.5. Patient 8

**Figure 2:**
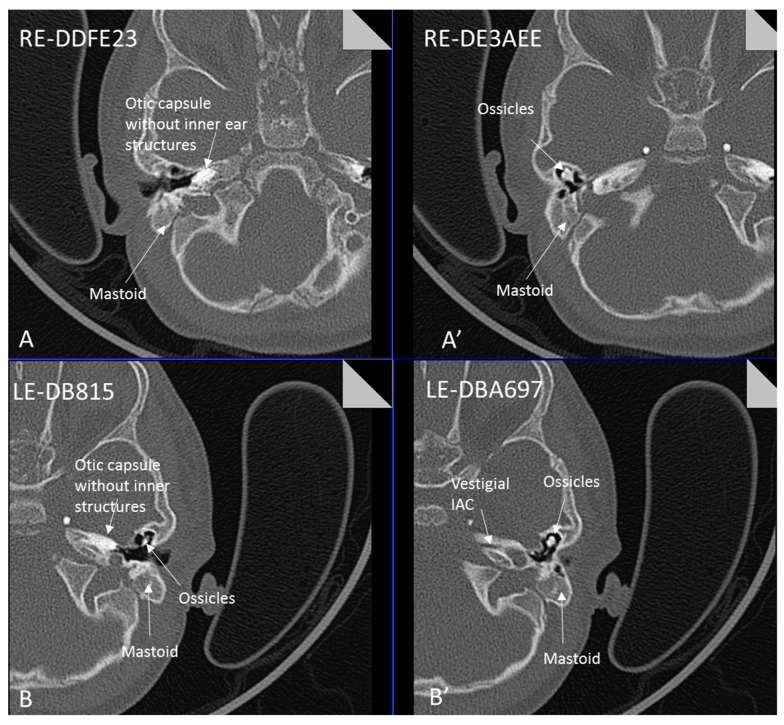
Axial CT petrous bone in patient 8 of the right (**A**,**A′**) and left (**B**,**B′**) ears shows complete labyrinthine aplasia (Michel deformity) characterised by absence of the inner ear structures: cochlea, vestibule, semicircular canals and vestibular and cochlear aqueducts. The otic capsule is hypoplastic and only a vestigial IAC is observed in the left ear (**B′**). Middle ear ossicles are bilaterally present and normal.

Patient 8 was a 1 year old daughter at the time of this study. She was the first child of nonconsanguineous Spanish parents and has a healthy sister. She was delivered normally at 42 weeks and failed newborn hearing screening (negative brainsteam evoked response audiometry, BERA), hence she was found to be profoundly deaf. She presented with psychomotor delay and she did not walk at the age of 1 year and 6 months. CT of petrous temporal bones (Figure 2) showed bilateral Michel’s aplasia with no visible inner ear structures. Only a vestigial internal auditory canal (IAC) is observed in the left ear (Figure 2B’). She has bilateral cupshaped ears, a tiny preauricular skin hillock on the left ear and small, widely-spaced teeth. She is compound heterozygous for a novel mutation c.173T>C (p.Leu58Pro) and c.283C>T (p.Arg95Trp), previously identified [9,11]. Her father and mother, obligate carriers of the p.Leu58Pro and p.Arg95Trp, respectively, and her sibling, carrier of the p.Leu58Pro mutation, did not show any clinical manifestation related to LAMM syndrome.

Two further patients with unilateral cochlear malformations were tested and found to be negative for *FGF3* mutations. A 7-year old Caucasian boy with unilateral profound hearing loss and short stature had absent left cochlea and semicircular canals on MRI; the right side was normal. A 3-year old Bengali boy with left-sided profound sensorineural deafness and normal right-sided hearing had a left sided facial skin tag and multiple pre-auricular skin tags. MRI of the inner ear showed a left common cavity, mildly dysplastic semi-circular canals and failure of the vestibulocochlear nerve to divide. The right side was normal. He had multiple hyperpigmented skin patches.

## 4. Discussion

Our patients exhibited a phenotypic spectrum from fully penetrant LAMM syndrome with complete inner ear agenesis (patients 1, 2, 5, 8) (Patient 8: Figure 2), to those with some rudimentary otocyst formation (patient 4, 6), incomplete partition type 1 on one side and a rudimentary otocyst on the other (patient 7), and mild external ear features with a mild dental phenotype (patient 3).

**Figure 3:**
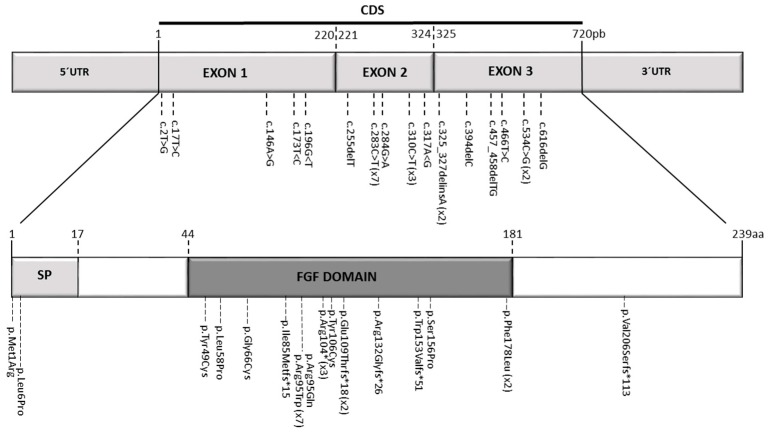
Mutational spectrum in the human FGF3 gene responsible for LAMM syndrome and its correlation at protein level. The mutation notation at DNA level is referred to the coding sequence (CDS, position 1 to 720), thus the position +1 corresponds to the adenine of the initiation codon (ATG) and the position 720 corresponds to the guanine in the last position of the stop codon (TAG). 5′UTR = 5′ Untranslated region in exon 1; 3′UTR = 3′ Untranslated region in exon 3; SP = signal peptide (1 to 17aa); FGF domain (44 to 181aa). Number coordinates and protein domains were obtained from the NCBI reference sequences NM_005247.4 (nucleotide) and NP_005238 (protein), respectively.

Three novel mutations were identified (Figure 3). The c.325_327delinsA (p.Glu109Thrfs*18) mutation is predicted to cause a frameshift resulting in premature termination of FGF3 protein. However, since this mutation affects the first three bases of the final coding exon of *FGF3*, an effect on splicing cannot be excluded, and the mutant transcripts may escape nonsense mediated mRNA decay. The c.284G>A (p.Arg95Gln) has also not previously been reported in the literature, but affects a well-conserved amino acid in a known functional domain of *FGF3*. However, mutation of the same codon c.283C>T (p.Arg95Trp), observed in cases 3, 4, 5, and 6, has previously been reported [9,11] and c.284G>A is therefore considered likely to be pathogenic. Both variants are present at low frequency in the gnomAD database [16], consistent with rare pathogenic variants in an autosomal recessive gene. The c.283C>T (p.Arg95Trp) mutation, has previously been reported to cause a milder phenotype with nearly normal looking auricles, variable inner ear structural phenotypes and milder dental phenotypes [11]. The present report provides further evidence for this. However, there is significant variation even within Family 3, with patient 5 having complete Michel’s aplasia and his two sisters having some rudimentary otocyst formation unilaterally. Finally, the novel c. 173T>C (p.Leu58Pro) identified in the Spanish family has also not been reported so far in the literature. It also affects a well-conserved amino acid position in a functional domain of FGF3 (Figure 3) and it has not been detected in control subjects at ExAC and Collaborative Spanish Variant Server-CSVS databases [17,18]. Interestingly, this mutation appears in compound heterozygosis with the previously described p.Arg95Trp mutation [9,11] in a patient displaying fully penetrant LAMM syndrome-congenital deafness, bilateral inner ear agenesis, microtia, microdontia and psychomotor retardation—further illustrating the variable expressivity and lack of penetrance resulting from *FGF3* mutations.

Mice lacking *Fgf3* have normal otic vesicle formation but may have abnormalities of dorsally derived structures, such as the endolymphatic duct and sac, incomplete partition and reduced coiling of the cochlear duct, and failure of complete separation of the anterior and posterior semicircular canals. However, development in mice in the absence of *Fgf3* is far more advanced than that generally observed in LAMM syndrome patients [6]. In mice, only double mutants lacking both *Fgf3* and *Fgf10* (a signalling molecule expressed in the mesenchyme underlying the otic placode) fail to form otic vesicles, indicating that in mouse at least, there is considerable functional redundancy between these two factors [4,5]. Sectioning of the Fgf3−/+, Fgf10−/+ double heterozygous mutant embryos showed that some had bilateral ‘microvesicles’ and that others had a unilateral microvesicle or none at all. Overall, 50% of mice showed a microvesicle, indicating phenotypic variability [4]. Mice lacking three of four FGF3/FGF10 alleles still formed otic vesicles, albeit smaller than controls or single homozygous mutant embryos (Fgf3−/− or Fgf10−/− embryos), with Fgf3−/+, Fgf10−/− being less severely affected, indicating a gradient of severity.

The reason for inter- and intrafamilial variability in human patients with LAMM syndrome is unknown but one can hypothesize similar effects to those observed in mouse studies with some possible functional redundancy between FGF signalling molecules, albeit less apparent than in the mouse. Furthermore, it is by no means established that mutations associated with LAMM syndrome act by a loss of function mechanism and so variable residual function attributable to different mutations may also be affecting severity of LAMM at least between families. Although some of the truncating and frameshifting mutations may appear to be loss-of-function, individuals with heterozygous complete gene deletion of *FGF3* have been described with Otodental syndrome [19], consisting of high frequency hearing loss and large teeth, inherited in a dominant fashion. Since parents of patients with LAMM syndrome are phenotypically normal, it may be that, while appearing to be compatible with loss-of-function these mutations may instead be hypomorphic.

Given the phenotypic variability observed between patients, and even between ears in the same patient, we investigated whether an extreme form of variability, a unilateral absent cochlea, with normal ear development on the contralateral side might also be caused by *FGF3* mutations. However, screening of two further patients with unilateral absent cochleae, indicating developmental arrest before the fourth week [20], failed to demonstrate *FGF3* mutations on Sanger sequencing even though the finding of *FGF3* mutations in Patient 7 with Incomplete Partition type 1, indicates a later developmental arrest at five weeks. As only Sanger sequencing was undertaken on DNA extracted from blood, mosaicism was not completely excluded and remains possible.

## Figures and Tables

**Table 1:** Clinical details of the eight patients presented in this work.

ID	Mutation	Right Ear	Left Ear	Teeth	Other Images	MRI Results	Interesting or Unusual Features
1	p.Glu109Thrfs*18 c.325_327delinsA homozygous	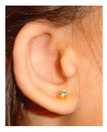	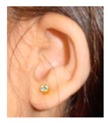	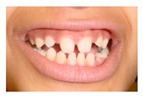	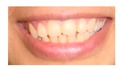 (Mother’s teeth; heterozygous)	Michel’s aplasia	Novel mutation; sacral pit; asymmetrical kidney size
2	p.Glu109Thrfs*18 c.325_327delinsA homozygous	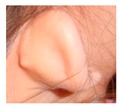	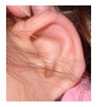	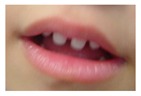		Michel’s aplasia	Novel mutation; possible social communication disorder
3	p.Arg95Trp c.283C>T homozygous	Normal	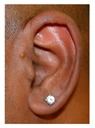	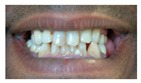		CT: Michel’s aplasia	Mild external ear and dental phenotype
4	p.Arg95Trp c.283C>T homozygous	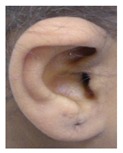	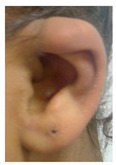	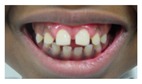		Michel’s aplasia (R); Minimal cochlear rest (L)	Large arachnoid cyst (R); asymmetrical kidney size; type 1 diabetes
5	p.Arg95Trp c.283C>T homozygous	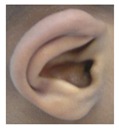	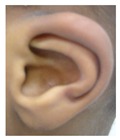	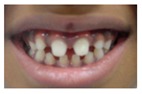		Bilateral Michel’s aplasia	
6	p.Arg95Trp c.283C>T homozygous	Not available	Not available	Not available		Michel’s aplasia and rudimentary otocyst (R)	Rudimentary otocyst with possible inner ear development (previously reported)
7	p.Arg95Gln p.Phe178Leu	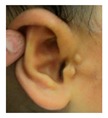	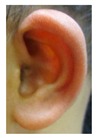	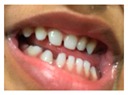	Parents teeth (both heterozygous): 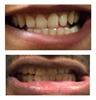	Cystic cochleo-vestibular malformation (R); cochlear aplasia with a rudimentary vestibule (L).	Novel mutation; compound heterozygote; milder phenotype of external ears without microtia
8	p.Leu58Pro p.Arg95Trp	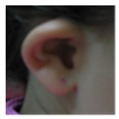	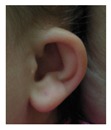	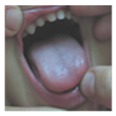		Michel’s aplasia	Novel mutation; compound heterozygote;

**Table 2:** Comparison of the phenotypic findings and mutations of our eight patients with previously reported LAMM cases.

	Current Report								Tekin 2007			Tekin 2008		Ramsebner 2010	Alsmadi 2009	Riazuddin 2011			Sensi 2011		Schaefer 2014	Singh 2014	Basdemirci 2019
	1	2	3	4	5	6	7	8	Fam A n = 5	Fam B n = 1	Fam C n = 3	Fam1 n = 1	Fam2 n = 3	Fam1 n = 4	Fam1 n = 22	Fam1 n = 4	Fam2 n = 7	Fam3 n = 3	Fam1 n = 2	Patient B	Fam1 n = 2	1	Fam1 n = 2
Ethnicity	Indian	Indian	Yemeni	Somalian	Somalian	Somalian	Indian	Spanish	Turkish			Turkish		Somali	Saudi	Pakistani	Pakistani	Pakistani	Albanian	Italian	French	Indian	Turkish
Consanguinity	+	+	+	+	+	+	-	-	+	+	+	+	+	Unknown	++	++	++	+	-	-	+	-	++
Profound congenital SNHL	+	+	+	+	+	+	+	+	+	+	+	+	+	+	+	+	+	+	+/-	+	+	+	+
Vestibular function	-	-	-	?	?	?	-	?	-	-	-	?	-	?	?	?	?	?	-	-	?	?	-
Mutation	c.325_327delinsA	c.325_327delinsA	c.283C>T	c.283C>T	c.283C>T	c.283C>T	c.284G>A c.534C>G	c.173T>C c.283C>T	c.466T>C	c.616delG	c.310C>T	c.255delT	c.17T>C	c.283C>T	c.196G >T	c.310C>T	c.283C>T	c.394delC	c.317A>G c.457_458 delTG	c.146A>G c.310C>T	c.2T>G	c.534C>G	c.196G>A
Protein effect	p.Glu109Thrfs*18	p.Glu109Thrfs*18	p.Arg95Trp	p.Arg95Trp	p.Arg95Trp	p.Arg95Trp	p.Arg95Gln p.Phe178Leu	p.Leu58Pro p.Arg95Trp	p.Ser156Pro	p.Val206Serfs*113	p.Arg104*	p. Ile85Metfs*15	p.Leu6Pro	p.Arg95Trp	p.Gly66Cys	p.Arg104*	p.Arg95Trp	p.Agr132Glyfs*26	p.Tyr106Cys p.Trp153Valfs *51	p.Tyr49Cys p.Arg104*	p.Met1Arg	p.Phe178Leu	p.Gly66Ser
External ear anomaly	+	+	+	+	+	+	+	+	+	+	+	+	+	+/-	+	+	+ (Mild)	+	+	+	+	+	+
External ear Symmetry	S	S	A	A	S	A	A	S	S	S	S	S	S	A	A	S	S	S	S	S	S	S	A
External ear details	T1-MO Bilat preauricular skin tag, cupped pinna	Cup shape, simple, protruding	Tiny preauricular skin hillock on one ear, no microtia	Cup shaped ears	Lop ear, T1M0	Lop ears	Large ears, skin tag on R.	Microtia+ Bilat. cupshaped ears, a tiny preauricular skin hillock on the left ear	T1-MO	T1-MO	T1-MO	T1-MO	T1-MO	Varied phenotype from normal auricles to T1-MO	T1-MO and anteverted ears	T1-MO	Some with near normal auricles; mild	T1-MO	T1-MO, anteverted ears, creased ear lobe.	T1-M0	T1-M0	T1-MO	Skin tag left auricule (n = 1) Shortened upper part auricules (n = 2)
Inner ear MRI features	Michels	Michels	Michels	L- otocyst rudimentary, R- Michel aplasia	Michels	L- Michel aplasia R- otocyst rudimentary	L- cystic vestibule R- cystic cochleovestibular malf	Michels, normal middle ear development	Michels	Michels	Michels	Michels	L- Michels R- rudimentarl cystic vestibule	Two subjects with rudimentary inner ear formation unilaterally	L- rudimentary vestibular structures R- Michels	Michels, normal middle ear development	Two subjects with cochlear basal turn, vestibule and posterior semi-circular canal	Michels, normal middle ear development	Middle ear involved. Hypoplasia petrous pyramids, bilateral labyrinth and IAC dysplasia. Stapes present in female.	CT Michels aplasia bilaterally Otic vesicle remnant ectopic in left mastoid	Michels; Middle ear structures involved	Michels	Michels
Inner ear (a)symmetry	S	S	S	A	S	A	A	S	S	S	S	S	A	A	A index	S	A n = 2	S	S	S	S	S	A
Dental anomalies	+	+	+	+	+	+	+	+	+	+	+	+	+	+	+	+	+ (mild)	+	+	+	+	+	+
Dental details SWS = Small, widely spaced	SWS	SWS	Mild	SWS gum hypertrophy	SWS	Microdontia	SWS	Microdontia	SWS	SWS	SWS			Microdontia, conical teeth		SWS	Mild	SWS	SWS	SWS	Persistent deciduous molars, dyschromia, enamel pits, taurodontism, oligodontia	Microdontia, thinned enamel, enlarged pulp	Hypodontia or oligodontia
Additional features	Renal size disparity	Autism	Sinus surgery	Gum HT, renal size disparity, long sighted	Long sighted	OSA, Squint	Motor delay	Motor delay	Unilateral stenosis of ureteropelvic junction (n = 1)	Strabismus and hypermetropia (n = 1)					Prominent nose tip, anteverted ears				Some perception loud sounds (n = 1); Occipital bone flattening, hypoplasic alae nasi	Carrier mother had unilateral ear defect surgically corrected.	Dextrorotation of heart (n = 1)	Hypophosphatemic rickets, language delay	Myopia, astigmatism, dilated azygos vein

Cases are detailed from [2,8,9,10,11,12,13,14,15]. A—Asymmetrical, S—Symmetrical, T1M0—Type 1 Microtia, SWS—Small widely spaced, Fam-Family.
